# Construction of Recombinant *Lactococcus lactis* Strain Expressing VP1 Fusion Protein of Duck Hepatitis A Virus Type 1 and Evaluation of Its Immune Effect

**DOI:** 10.3390/vaccines9121479

**Published:** 2021-12-14

**Authors:** Xiaoting Zhang, Ruihua Zhang, Jingyu Wang, Nana Sui, Guige Xu, Hui Yan, Yanli Zhu, Zhijing Xie, Shijin Jiang

**Affiliations:** 1 Department of Preventive Veterinary Medicine, College of Veterinary Medicine, Shandong Agricultural University, Taian 271018, China; xtzhang2021@126.com (X.Z.); rhzhang@sdau.edu.cn (R.Z.); jywang676@163.com (J.W.); sn17763223251@163.com (N.S.); sdxuguige@163.com (G.X.); 17863805800@163.com (H.Y.); ylz@sdau.edu.cn (Y.Z.); xiezhj@sdau.edu.cn (Z.X.); 2 Shandong Key Laboratory of Animal Microecological Preparations, Taian 271000, China; 3 Shandong Provincial Key Laboratory of Animal Biotechnology and Disease Control and Prevention, Taian 271000, China

**Keywords:** duck hepatitis A virus type 1, VP1 protein, *Lactococcus lactis*, mucosal immune

## Abstract

With the continuous development of duck farming and the increasing breeding density, the incidence of duck hepatitis A virus type 1 (DHAV-1) has been on the rise, seriously endangering the development of duck farming. To reduce the use of antibiotics in duck breeding, susceptibility risks and mortality, and avoid virulence recovery and immune failure risk, this study aims to develop a new type of mucosal immune probiotics and make full use of molecular biology techniques, on the level of genetic engineering, to modify *Lactococcus lactis* (*L. lactis*). In this study, a secretory recombinant *L. lactis* named MG1363-VP1 with an enhanced Green Fluorescent Protein (eGFP) and translation enhancer T7g10L was constructed, which could express the VP1-eGFP fusion protein of DHAV-1. The animal experiment in ducklings was performed to detect the immune response and protection effect of oral microecologics by recombinant *L. lactis*. The results showed that oral *L. lactis* MG1363-VP1 significantly induced the body’s humoral immune system and mucosal immune system to produce specific anti-VP1 IgG antibodies and mucosal secretory immunoglobulin A (sIgA) for DHAV-1 in ducklings, and cytokines including interleukin-2 (IL-2), interleukin-4 (IL-4), interleukin-10 (IL-10), and interferon gamma (IFN-γ). The mortality rate was monitored simultaneously by the natural infestation in the process of production and breeding; notably, the ducklings vaccinated with *L. lactis* MG1363-VP1 were effectively protected against the nature infection of DHAV-1. The recombinant *L. lactis* MG1363-VP1 constructed in this study provides a new means of preventing and controlling DHAV-1 infection in the future.

## 1. Introduction

Duck virus hepatitis (DVH) is a highly fatal and rapidly spreading contagious disease in ducklings that is mainly caused by duck hepatitis virus (DHV). The three DHV types are all RNA viruses: duck hepatitis A virus (DHAV), duck astrovirus type 1 (DAstV-1), and duck astrovirus type 2 (DAstV-2), respectively [1]. The DHAV is genetically divided into three serotypes: DHAV type 1 (DHAV-1), type 2 (DHAV-2), and type 3 (DHAV-3) [2,3,4,5]. DHAV-1 is common and widely distributed in ducks, especially ducklings < 3 weeks old, as it can cause 100% morbidity. The typical pathological changes are liver hemorrhage and swelling [6]. DHAV-1 belongs to the genus *Avihepatovirus* of the family *Picornaviridae* [7]. The capsid protein of the virus encapsulates the main genetic material, the capsid proteins of DHAV-1 include VP0, VP1, and VP3. VP1 was considered the external and dominant antigen with several conserved linear epitopes. VP1 protein has the highest genetic diversity amongst the different isolates and can induce neutralizing antibodies in ducks [8].

The mucosal immune system is an important part of the body’s immune system, which plays an important role in resisting infection. Most pathogens enter the body through mucosal surfaces, and the development of a vaccine that is protective at such sites should be very effective. The mucosal tissue of the gastrointestinal tract is the main place for local specific mucosal immunity [9]. Lactic acid bacteria (LAB) play a key role in maintaining the intestinal balance [10]. LAB have proved to be effective mucosal delivery vehicles that overcome the problem of delivering functional proteins to the mucosal tissues [11]. LAB live carrier immune microecological preparation has the following advantages: it can stimulate the body’s mucosal immune system and activate mucosal immune response. Compared with the traditional intramuscular injection of vaccines, it can reduce the stress reaction of animals. As an antigen, the foreign protein expressed by LAB can increase specific IgG antibodies, which can neutralize pathogens and delay infection. Several delivery systems have been developed to target heterologous proteins to a specific cell location (cytoplasm, cell wall, or extracellular medium) and, more recently, to efficiently transfer DNA to eukaryotic cells [12]. At present, a variety of exogenous proteins have been successfully constructed and expressed in LAB. Mucosal immune probiotics have attracted increasing attention.

As a food-grade LAB, *Lactococcus lactis* (*L. lactis*) has been extensively used as a delivery vehicle for oral vaccines. In this study, we constructed a continuous secretory expression system with *L. lactis*. The recombinant *L. lactis* MG1363-VP1, which could express the recombinant protein of DHAV-1/VP1, can be used as an oral vaccine for prevention and control of DHAV-1 infection.

## 2. Materials and Methods

### 2.1. Bacterial Strain and Vector

The DHAV-1 strain LY0801 (accession no. FJ436047), pR-DHAV-1 infectious clone and 4F8 monoclonal antibodies (mAb) were all made in our previous work [13]. The plasmid pMG36e and *L. lactis* strain MG1363 were purchased from BIO SCI BIO (Hangzhou, China). The *Escherichia coli* (*E. coli*) DH5α competent cells was purchased from TaKaRa (Dalian, China). The plasmids pMD18-T and pEGFP-C3 were purchased from TransGen Biotech Co., Ltd. (Beijing, China).

### 2.2. Main Reagents and Antibodies

The 2 × Phanta Master Mix and Clone Express II One Step Cloning Kit were purchased from Nanjing Vazyme biotech Co., Ltd. (Nanjing, China). The PurePlasmid Mini Kit and Gel Extraction Kit were both purchased from Beijing Cowin Biotech Co., Ltd. (Beijing, China). The dialysis bag and lysozyme were purchased from Beijing Solarbio Science and Technology Co., Ltd. (Beijing, China). The Express Cast PAGE, 5 × SDS-PAGE Loading Buffer and Ncm Ecl Ultra were purchased from New Cell Molecular Biotech. The enhanced HRP-DAB Chromogenic Kit was purchased from Tiangen biotech. The HRP conjugated goat anti-mouse IgG was purchased from Sigma. The HRP-conjugated goat anti-duck IgG was purchased from KPL (Gaithersburg, MD, USA). The DHAV-1 egg yolk antibody named as Yagankang was purchased from Tianjin Ringpu Biotechnology Co., Ltd. (Tianjin, China), and the neutralizing antibody titers of the antibody against DHAV-1 was higher than the titer of 1: 512. The duck IL-2 enzyme linked immunosorbent assay (ELISA) kit, sIgA ELISA kit, IFN-γ ELISA kit, IL-4 ELISA kit, and IL-10 ELISA kit were all purchased from Shanghai MLbio Biotechnology Co., Ltd. The *L. lactis* MG1363 was cultured in M17 medium supplemented with 0.5% glucose (GM17, Haibo, Qingdao, China). The erythromycin was purchased from Beijing Solarbio Science and Technology Co., Ltd. (Beijing, China), the working concentration of which was 200 μg/mL in DH5α and 2 μg/mL in *L. lactis*.

### 2.3. Expression Plasmid Construction in E. coli

**Figure 1:**
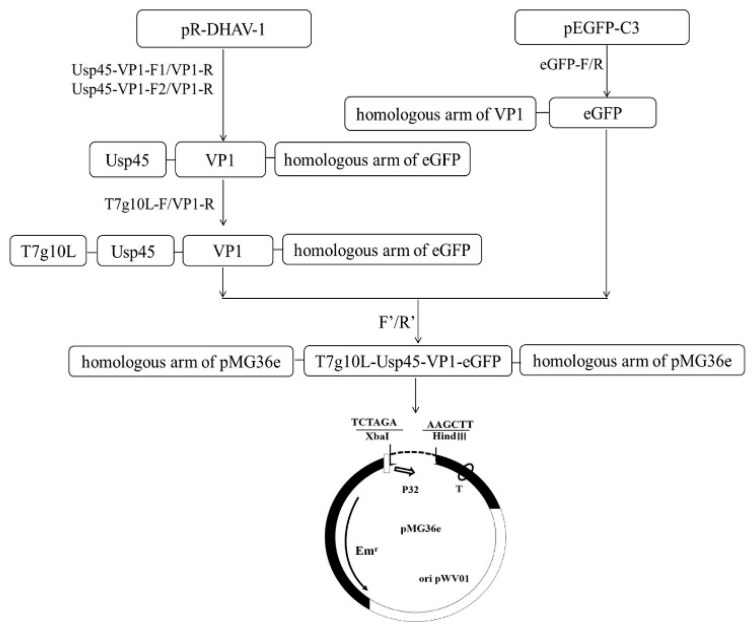
Construction diagram of the recombinant plasmid. The fused T7g10L-Usp45-VP1-eGFP was finally inserted into the plasmid pMG36e after being digested by *Xba* I and *Hind* III.

The primers used in this study are listed in Table 1. Using plasmid pR-DHAV-1 as template, the first fragment of 781 bp was amplified with primers Usp45-VP1-F1/VP1-R. The second fragment of 818 bp, with an 81 bp signal peptide, was amplified using the first fragment as template with primers Usp45-VP1-F2/VP1-R. Using gel extract production in the second step as the template and T7g10L-F/VP1-R as primers, an 840 bp fusion gene T7g10L-Usp45-VP1 with homologous arm of eGFP was obtained. Using plasmid pEGFP-C3 as template, a fragment of 816 bp with homologous arm of VP1 was amplified with primers eGFP-F/eGFP-R. The T7g10L-Usp45-VP1 and eGFP were fused together with primers T7g10L-F/eGFP-R and linked to the vector pMD18-T to be sequenced. Then, the homologous arm of vector pMG36e was added to the T7g10L-Usp45-VP1-eGFP with primers F’/ R’ and the final 1612 bp fragment was inserted to plasmid pMG36e with the restriction enzyme *Xba* I and *Hind* III. The recombinant plasmid was transformed into the *E. coli* DH5α competent cells, and the positive clones were sequenced by Sangon Biotech Co., Ltd. (Shanghai, China) (Figure 1).

### 2.4. Construction of Recombinant L. lactis MG1363-VP1

The competent cells of *L. lactis* MG1363 were prepared in advance. The Usp45-VP1-eGFP-pMG36e positive plasmid was transferred into the competent cells by electroporation technology [14], and the cells were cultured in GM17 agar medium containing erythromycin at a concentration of 1 μg/mL. The recombinant plasmids were extracted and identified by polymerase chain reaction (PCR) and enzyme digestion. The positive plasmid was transformed into the *L. lactis* MG1363 for the second time. According to the 16S rRNA specific sequence of *L. lactis* MG1363 published in GenBank, a pair of specific primers was designed (Table 1). The *L. lactis* strain was identified by Gram staining and the 16S rRNA sequencing. The positive recombinant MG1363 strain contain Usp45-VP1-eGFP-pMG36e, renamed as MG1363/VP1. At the same time, the empty vector pMG36e was electroporated into *L. lactis* MG1363 (renamed as MG1363/pMG36e) as a negative control.

### 2.5. Fluorescence Microscopy

An appropriate amount of recombinant *L. lactis* solution was taken on sterile slides, dried, and then covered with glass slides for observation under the Nikon upright fluorescence microscope 55i (Nikon, Japan).

### 2.6. Western Blot Analysis

The recombinant *L. lactis* MG1363/VP1 and MG1363/pMG36e were grown in GM17 without erythromycin and cultured at 37 °C for 10–16 h. The culture supernatant and the bacterial pellet sedimentation were separated by centrifugation at 12000 rpm for 5 min.

The supernatant was filtered through a 0.22 μm pore size membrane and concentrated by ammonium sulfate precipitation (50%) in dialysis bag. The obtained protein precipitation was washed by bacterial lysis buffer. The intracellular protein was purified as follows: the bacteria pellets were washed by adding 1/10 volume of bacterial lysis buffer and lysozyme (20 mg/mL). After being incubated at 37 °C for 60 min, the pellets were broken by ultrasound under ice bath conditions with a 100 W power. The ultrasonication procedure was 45 cycles of ultrasound for 5 s and interval for 8 s. After ultrasound, the supernatant was collected by centrifugation at 12000 rpm for 10 min. The inclusion protein was purified as follows: the sedimentation was washed with washing buffer (50 mM/L Tris-HCl, 100 mM/L NaCl, 10 mM/L EDTA, 0.5% Triton X-100) at different urea concentrations (0 M, 2 M, 4 M, 8 M) successively.

A following Western blot assay was designed to detect if the protein existed in the supernatant or intracellular. The DHAV monoclonal antibody 4F8 and the HRP-conjugated goat anti-mouse IgG (Sigma, 1:4000) were respectively used as the primary antibody and secondary antibody to detect the DHAV-1 VP1 protein as described previously [12].

### 2.7. Intestinal Colonization of Recombinant L. lactis

A total of 10 one-day-old cherry valley ducklings were bought from Jingwei husbandry company and were orally immunized with the recombinant *L. lactis* MG1363/VP1 at three or four days of age. Each duckling was orally immunized by one milliliter (1 × 10^9^ CFU/mL) and slaughtered at 15 days. Smears were randomly sampled from each segment of the intestinal mucosal epithelium for the duodenum, jejunum, ileum, cecum, colon, and observed under a fluorescent microscope with a cover glass.

### 2.8. Oral Vaccination of Ducklings

**Figure 2:**
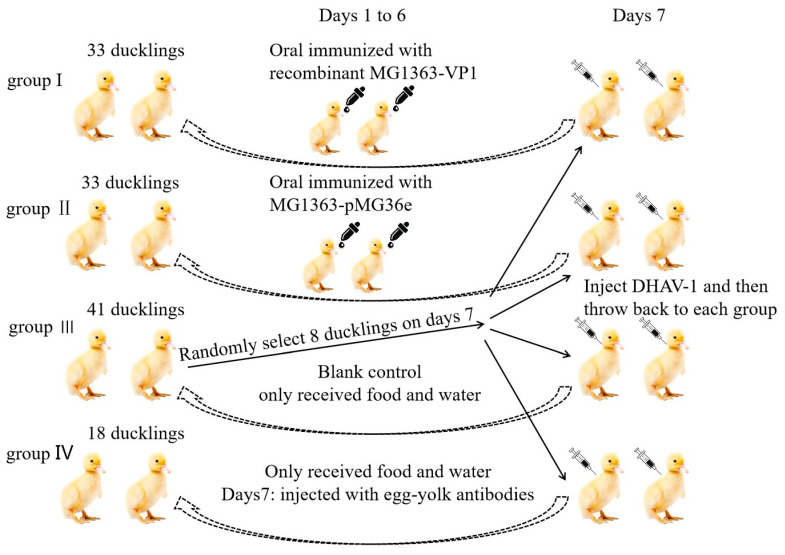
Oral vaccination experiment in ducklings. A total of 125 one-day-old ducklings were randomly divided into four groups, named group I (MG1363-VP1, 33 ducklings), group II (MG1363-pMG36e, 33 ducklings), group III (blank control group, 41 ducklings), and group IV (egg-yolk antibody group, 33 ducklings).

A total of 130 one-day-old cherry valley ducklings were bought from Jingwei husbandry company, Taian city. Five one-day-old ducklings were randomly slaughtered to collect serum and intestine samples. The remaining ducklings were divided into four groups, named group I (33 ducklings), group II (33 ducklings), group III (41 ducklings), and group IV (18 ducklings). Ducklings in group I and group II were orally immunized with *L. lactis* MG1363-VP1 and *L. lactis* MG1363-pMG36e, respectively, from day 1 to day 6. Each duckling in the two groups was orally immunized with 1 mL (1 × 10^9^ CFU/mL). The five ducklings were randomly slaughtered at 3, 5, and 7 days, after 48 h, 96 h, and 134 h of oral immunization, to collected serum and duodenum. Group III were given food and water only as a blank control group and were also slaughtered at the same time. Ducklings in group IV only received food and water and were then injected with egg-yolk antibodies (1 mL/duckling) at 7th day. Finally, on the day 7, 8 ducklings were randomly selected from group III to infect with DHAV-1 (strain LY0801) through the eyes and nose (0.3 mL/duckling; 2 × 10^4^ copies). Next, each group was put in 2 ducklings infected with DHAV-1 to mimic natural infection (Figure 2). From day 8, the surviving number of ducklings in each group was recorded.

### 2.9. Detection of Cytokines and Specific Antibody in Ducklings

The 96-well plate was coated with purified VP1-eGFP protein, and the optimal dilution concentration and dilution ratio of protein were determined in advance using the Dot Matrix method. Finally, the optimal protein coating concentration was 7.5 μg/mL; the 96-well plate is coated with this concentration, 100 μL/well, incubated at 4 °C overnight. HRP-conjugated goat anti-duck IgG (KPL, 1:5000 dilution) was used to detect duck blood serum. The intestine fluids of ducklings were examined by sIgA commercial ELISA kit. The cytokines IL-2, IL-4, IL-10, and IFN-γ in blood-serum samples were detected using commercial ELISA kits according to the manufacturer’s instructions.

## 3. Results

### 3.1. Construction of Recombinant Plasmid Usp45-VP1-eGFP-pMG36e

The full length of the plasmid Usp45-VP1-eGFP-pMG36e was 5204 bp; the restriction site *Sac* I was found at 1566 bp and 2692 bp. The suitable restriction site *Sac* I in the plasmid was selected for single restriction. After digestion, two bands of about 3633 bp and 1571 bp were obtained. The plasmid was confirmed to have no amino acid mutations after sequencing. 

### 3.2. Construction of Recombinant L. lactis MG1363-VP1

**Figure 3:**
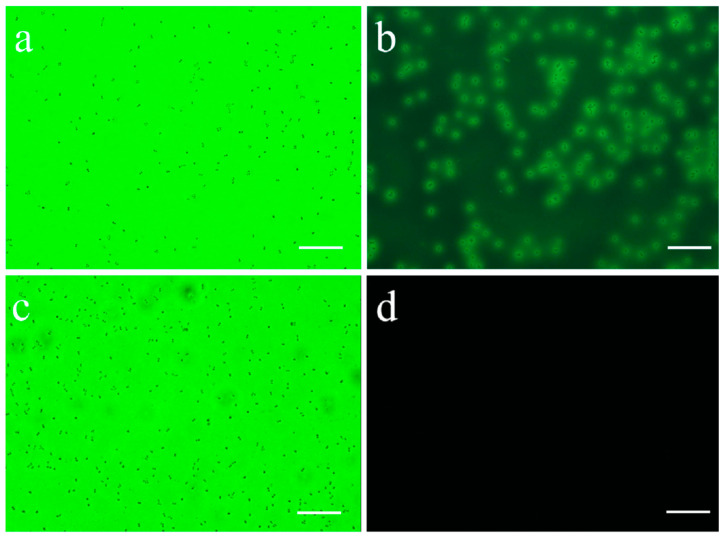
The results of fluorescence microscopy: (**a**) bright field of MG1363-VP1; (**b**) fluorescent field of MG1363-VP1; (**c**) bright field of MG1363-pMG36e; (**d**) fluorescent field of MG1363-pMG36e. (Scale size: 20 μm).

The recombinant plasmid Usp45-VP1-eGFP-pMG36e was introduced into *L. lactis* MG1363 competent cell by the electro-transfer method. The conditions were a pulse of 25 μf, voltage of 2200 V, and resistance of 200 Ω. The positive recombinant *L. lactis* was screened in GM17 medium containing erythromycin. Single-colony strains were selected in culture. Fluorescence microscope showed that the recombinant *L. lactis* was positive (Figure 3).

**Figure 4:**
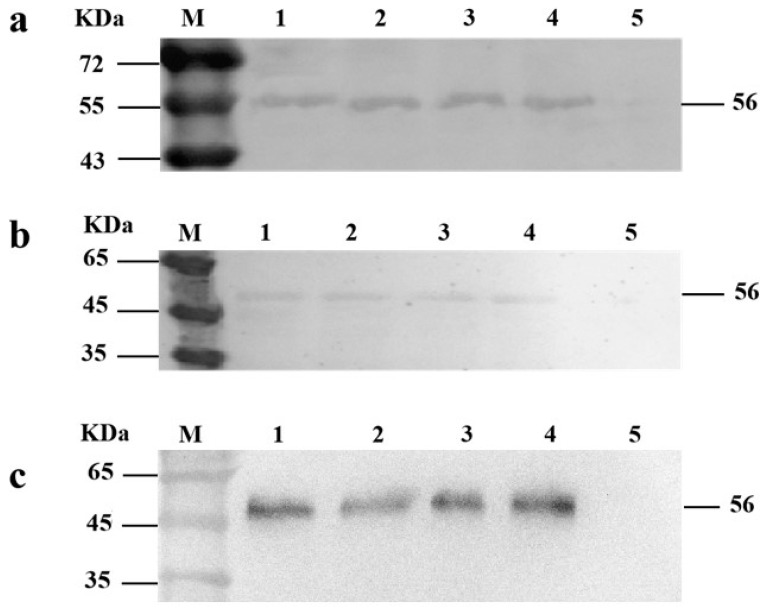
The results of Western blotting: M: maker; (**a1**) supernatant protein of 10 h; (**a2**) supernatant protein of 12 h; (**a3**) supernatant protein of 14 h; (**a4**) supernatant protein of 16 h; (**a5**) negative control; (**b1**) intracellular soluble protein of 10 h; (**b2**) intracellular soluble protein of 12 h; (**b3**) intracellular soluble protein of 14 h; (**b4**) intracellular soluble protein of 16 h; (**b5**) negative control; (**c1**) inclusion protein of 10 h; (**c2**) inclusion protein of 12 h; (**c3**): inclusion protein of 14 h; (**c4**) inclusion protein of 16 h; (**c5**) negative control.

We used Western blotting to detect the VP1-eGFP protein expression, using *L. lactis* MG1363-pMG36e as the negative control. The target protein in cells can be detected after 10–16 h culture. The protein exists in supernatant and intracellular, the intracellular protein exists in two forms, the soluble protein and insoluble protein (Figure 4).

### 3.3. Immune Response and Protection Effect in Ducklings after Oral Immunization

**Figure 5:**
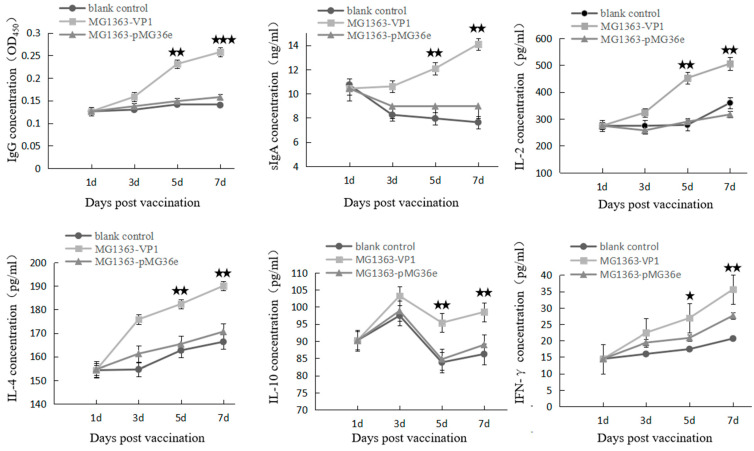
Detection of immune responses in serum or intestine samples of ducklings. ★ *p* < 0.05; ★★ *p* < 0.01; ★★★ *p* < 0.001.

The IL-2, IL-4, IL-10, IFN-γ, total sIgA, and anti-VP1 antibody were detected at 3, 5, and 7 days. One-way analysis of variance multiple range test in GraphPad prism5 was used to analyze data. Statistical significance of *p*-values < 0.05 was set (Figure 5). The concentrations of IgG, sIgA, IL-2, IL-4, and IFN-γ in ducklings of MG1363-VP1 were significantly higher than those in ducklings of MG1363-pMG36e and the blank control group on days 5–7. The concentration of IL-10 in MG1363-VP1 group rose to the peak at the third day; it then went down and rose again.

**Figure 6:**
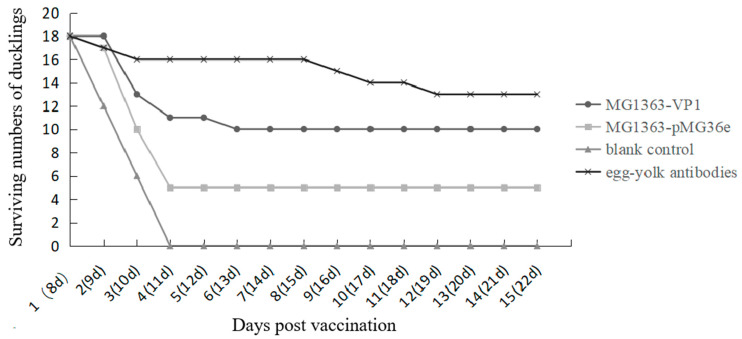
Surviving numbers of the ducklings after natural DHAV-1 infection.

The infection period in this study mimicked the natural infection for 15 days. All ducklings in the blank control group died on the fourth day after infection; the final mortality rate reached 100%. At the same time, the ducklings that were orally immunized with *L. lactis* MG1363-pMG36e died faster after infection; there were 13 deaths in 4 days, and the final survival rate was 27% (5/18). Different from the blank control group, the MG1363-pMG36 control group is partially protective. The ducklings that were orally immunized with *L. lactis* MG1363-VP1 no longer died after 6 days of infection, and the final protection rate was 55% (10/18). In the egg-yolk antibodies group, only two ducklings died within 4 days after infection, and there was no death thereafter, the final survival rate was 72% (13/18) (Figure 6).

### 3.4. The Intestinal Colonization of L. lactis

**Figure 7:**
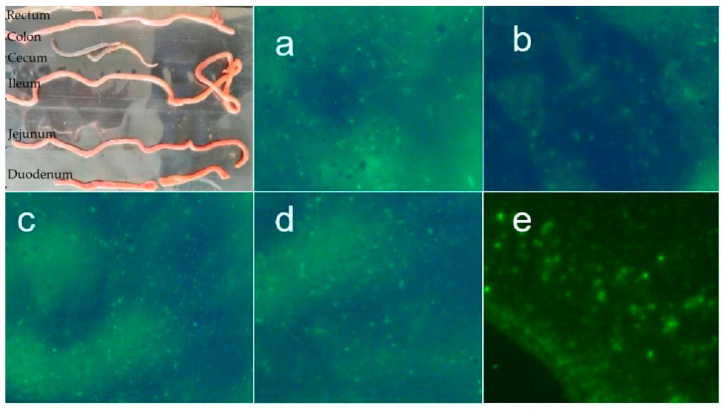
Fluorescence microscopy of intestinal mucosal epithelial contents: (**a**) duodenum; (**b**) jejunum; (**c**) ileum; (**d**) cecum; (**e**) colon.

Under fluorescence microscope, the *L. lactis* could be found in each segment of the intestine (Figure 7). The *L. lactis* colonization test proved that *L. lactis* could colonize in the intestine for at least 15 days.

## 4. Discussion

DHAV-1 cause high mortality in ducklings and pose a serious threat to duck farming. At present, the prevention and control of the disease mainly depend on live attenuated vaccines. However, to reduce the cost of breeding, the farms only use yolk antibody for emergency immunization when the duck population is found to be infected. Although a high protection rate can be obtained, a great deal of time and labor is wasted. It can also lead to immune failure. The use of LAB as a safe and efficient cell factory to produce heterologous proteins is of medical interest [15,16,17,18]. The use of a LAB strain in clinical trials in humans to alleviate inflammatory bowel diseases has opened the possibility of using LAB to target other diseases [19]. *L. lactis* strain MG1363 is recognized as a non-pathogenic, safe-grade food microorganism that can express foreign antigens [20], so we selected it in this study. In our earlier study, we constructed a recombinant *L. lactis* that could express a recombinant protein of DHAV-3/VP1 relying on the nisin-controlled inducible expression system [21]. However, the LAB-induced expression system needs to add nisin inducers to express the target protein. After the bacteria were lysed, the antigen protein is released. To avoid excessive accumulation and degradation of the target protein in the cell and maximize the secretion of recombinant protein into the extracellular space, the native signal peptide from a major secreted lactococcal proteins, Usp45, is used [22]. This study optimized the LAB expression system on this basis and selected the *E. coli*-LAB shuttle expression vector pMG36e. Under the control of native strong constitutive promoter P32, a signal peptide of the major secreted protein of *L. lactis*, Usp45 is fused to the N-terminus of the target protein VP1. This signal peptide has been confirmed in almost all secreted expression of LAB [23,24,25]. Studies into its secretion mechanism have shown that the precursor protein containing the signal peptide (SP) is formed first, and then the signal peptide is cleaved off by the cleaved enzyme during the process of passing through the cell membrane, and the mature protein is released [26]. We verified that the recombinant *L. lactis* not only expresses foreign proteins successfully, but also secretes the proteins into the supernatant; the protein exists as soluble and insoluble forms in the cell. We speculated that due to the introduction of the translation enhancer T7g10L, some intracellular proteins exist in the form of inclusion bodies. T7g10L is the leader sequence of gene 10 from the T7 phage, which has an obvious effect, promoting gene translation [27,28]. There are precedents in the application of LAB expression vectors [29,30]. The biological activity of the strain can be detected in each segment of the intestine after oral immunization for a period. We speculate that the recombinant strain can tolerate the environment of the duck’s gastrointestinal tract and can adhere and colonize the intestine for a period. Without erythromycin selection pressure, the stability rate of the expression vector pMG36e in *L. lactis* MG1363 was 96–100% [31]. Therefore, the recombinant *L. lactis* can be used in fermentation production without adding antibiotics, which has a certain practical value.

LAB has Toll-like receptor agonists, which can stimulate and activate immune cells, thereby enhancing antigen-specific immune response [32,33]. Toll-like receptor activates dendritic cells by transducing intracellular signals and the associated recognition of extracellular proteins and the information they carry to dendritic cells, providing acquired immuno-stimulatory signals, and then inducing the production of some pro-inflammatory cytokines, such as IL-12 and IFN-γ [34]. As a vaccine carrier, LAB does not have the pro-inflammatory factor-lipopolysaccharide of Gram-negative bacteria, nor does it produce a variety of miscellaneous proteins [35,36]. It mainly induces Th1 type immune response [37]. IL-2 and IFN-γ cytokines are associated with Th1 cell subpopulation and mediate cellular immunity. IL-4, IL-6, IL-10, and other cytokines are associated with Th2 cell subsets and mediate humoral immunity [38]. The results of animal immunoassay showed that the recombinant LAB microprobiotics with duck hepatitis A virus VP1 protein could significantly increase the content of cytokines in animal serum. Through the detection of various cytokines and specific antibodies through animal experiments, it was found that the LAB has good immunogenicity, and there are significant differences between each group of data. It activates the intestinal mucosal immune system and induces humoral and cellular immunity. What interests us is that the changing trends of IL-10 are different from other cytokines. IL-10, produced by a wide variety of cells, is a highly pleiotropic cytokine. Induction of IL-10 often occurs together with pro-inflammatory cytokines, although the pathways that induce IL-10 may negatively regulate these pro-inflammatory cytokines [39]. As the probiotic, *L. lactis* itself can improve the body’s resistance but usually not produce a strong immune response. Moreover, the IFN-γ concentration in MG1363-pMG36e immunized ducklings were higher than the non-immunized ducklings in Figure 5. Due to immunological effect of IFN-γ, some ducklings with relatively high concentration were protected from DHAV-1 infection during the susceptibility period. To date, LAB oral immune microecological immune preparations have received extensive attention and recognition. The DHAV-1 recombinant *L. lactis* with fluorescent groups constructed in this study has good biological activity and lays the foundation for subsequent in-depth research.

## 5. Conclusions

In summary, a recombinant *L. lactis* MG1363-VP1 was successfully constructed in this study, and it was proved that the fusion protein could be successfully continuously expressed in the recombinant *L. lactis* without induction. After the oral immunization, specific antibodies can be detected in ducklings, and the protection rate can reach 50% after DHAV-1 virulent infections.

## Figures and Tables

**Table 1:** Primers used in this study.

Primers	Sequences (5′ to 3′)
Usp45-VP1-F1	cagtgatactttctgctgcagccccgttgtcaggtgtttacgctGGTGATTCTAACCAGT
Usp45-VP1-F2	atgaaaaaaaagattatctcagctattttaatgtctacagtgatactttctg
VP1-R	aacagctcctcgcccttgctcaCTTCAATTTCCAAATTGAGTTC
T7g10L-F	TCTAGAaataattttgtttaactttaagATGAAAAAAAAGATTATCTCA
eGFP-F	AATTTGGAAATTGAAgtgagcaagggcgaggagctgttcac
eGFP-R	AAGCTTtcagttatctagatccggtggatcccgggcccgcg
F’	ctcgcccggggatcgatccTCTAGAaataattttgtttaactttaag
R’	accttcgttttcagactttgcAAGCTTtcagttatctagatccggtggatc
MG1363-F	GATGATACATAGCCGACCTGA
MG1363-R	TTAGCCGTCCCTTTCTGG
